# Ethnicity and cardiovascular mortality in Brazil: a call for papers: Etnia e mortalidade cardiovascular no Brasil: um convite para submissão de artigos

**DOI:** 10.1590/1516-3180.2015.13332904

**Published:** 2014-12-19

**Authors:** Paulo Andrade Lotufo

**Affiliations:** I MD, DrPH. Full Professor, Department of Internal Medicine, Faculdade de Medicina da Universidade de São Paulo (FMUSP), São Paulo, Brazil.

## THE CHALLENGE OF ETHNICITY/RACE/SKIN COLOR AND HEALTH OUTCOMES

Despite the consensus that age, sex, parental history, hypertension, dyslipidemia, smoking and diabetes are risk factors for cardiovascular diseases,^1^ race, ethnicity or skin color always bring an anthropological debate into biology.^2^ First, there are different views about the meaning of race according to culture and country. Second, the potential influence of genetic background is blurred by differential distribution of the proportions of formal education and income according to race/ethnicity/skin color. Third, considering that the foundation of racially admixed societies, such as most of the countries in the Americas, was based on subjugation of the indigenous populations and imported slavery of Africans, the presence of racism and discrimination cannot be neglected as one point in the causal pathway of cardiovascular diseases according to race/ethnicity/skin color. Fortunately, the emergence of molecular biology and genomics is opening up a new road towards understanding the influence of race on several diseases. Although the terms “race” and “skin color” are very popular, we will prefer “ethnicity” because it encompasses biology, culture, language and religion, thereby revealing health behavior and beliefs.^3^

## THE BRAZILIAN PATTERN OF CARDIOVASCULAR MORTALITY ACCORDING TO ETHNICITY

The Brazilian national census of 2010 was the first to assess self-reported race. In that year, 99.5% of the respondents completed the section on race/skin color. The census showed that 47.8% of the population self-reported as white, 43.1% mixed or brown, 7.6% black, 1.1% Asian and 0.4% indigenous. On the other hand, recording mortality on death certificates according to race or skin color was established in Brazil in 1996. The lowest proportion of leaving race blank on the death certificate form (6.4%) and the lowest proportion of deaths due to unknown causes (7.0%) occurred in 2010. Thus, 2010 was the best year for calculating death rates according to race without using predicted values for the population. Taking into consideration the aim of reducing premature mortality due to non-communicable diseases,^4^ we evaluated the age-adjusted cardiovascular death rate according to race in Brazil in 2010 (Table 1).

**Table 1.**
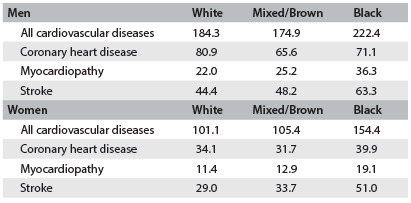
Age-adjusted death rates (per 100,000) according to ­self-declared ethnicity/race/skin color in Brazil (2010) for men and women aged 30 to 69 years

All deaths of individuals aged between 30 and 69 years that occurred in 2010 were classified using the International Classification of Diseases, Tenth Revision (ICD-10). The age group studied was chosen because it covered premature deaths. Those classified as Asian or indigenous were not included due to their small numbers. The numbers of deaths classified as being due to all types of cardiovascular disease (ICD-10: chapter I), and due to coronary heart disease ­(ICD-10: I20-25), myocardiopathy (ICD-10: I-11; I-42-43; I-50) and stroke ­(ICD-10: ­I-60-69), were calculated. All of the data were obtained from the archives of the Ministry of Health Mortality Information System. Population data were taken from the 2010 national census, collected by the Brazilian Institute for Geography and Statistics (IBGE). The rates (per 100,000 inhabitants) were adjusted for the Brazilian population using the direct method, and were presented according to sex and race/skin color. More details can be obtained elsewhere.^5^

For both sexes, all causes of death were higher among blacks, followed by whites and mixed/brown individuals. Black men and women had higher risks of death due to all cardiovascular diseases, myocardiopathy and stroke but not due to coronary heart disease. White and mixed/brown individuals presented similar rates for all categories, although the risk of death due to any cardiovascular disease was slightly lower for whites.^5^

## A CALL FOR PAPERS ADDRESSING ETHNICITY AND ADVERSE HEALTH OUTCOMES

The explanations for these findings may include the following: (1) misclassification of race on death certificates; (2) higher incidence of cerebrovascular disease and myocardiopathy among blacks and higher incidence of coronary heart disease among whites; and (3) the case-fatality rates of acute cerebrovascular and coronary events may be a differential factor between white, mixed/brown and black individuals. Our aim is not to discuss these possibilities in this editorial. The reason for raising this issue is that the *São Paulo Medical Journal* is opening a call for papers addressing ethnicity and adverse health outcomes in Brazil and elsewhere, especially in relation to societies with a high proportion of people with mixed ancestry. Submissions of manuscripts on this theme will be evaluated in a fast-track system.

We welcome a good debate about ethnicity and health.
